# Fault Detection and Diagnosis Using Combined Autoencoder and Long Short-Term Memory Network

**DOI:** 10.3390/s19214612

**Published:** 2019-10-23

**Authors:** Pangun Park, Piergiuseppe Di Marco, Hyejeon Shin, Junseong Bang

**Affiliations:** 1 Department of Radio and Information Communications Engineering, Chungnam National University, Daejeon 34134, Korea; 2 Department of Information Engineering, Computer Science and Mathematics, University of L’Aquila, 67100 L’Aquila, Italy; piergiuseppe.dimarco@univaq.it; 3 Dental Clinic Center, Kyungpook National University, Daegu 41940, Korea; shjrei@gmail.com; 4 Defense & Safety ICT Research Department, Electronics and Telecommunications Research Institute, Daejeon 34129, Korea

**Keywords:** autoencoder, long short-term memory, rare event, fault detection, fault diagnosis, time delay

## Abstract

Fault detection and diagnosis is one of the most critical components of preventing accidents and ensuring the system safety of industrial processes. In this paper, we propose an integrated learning approach for jointly achieving fault detection and fault diagnosis of rare events in multivariate time series data. The proposed approach combines an autoencoder to detect a rare fault event and a long short-term memory (LSTM) network to classify different types of faults. The autoencoder is trained with offline normal data, which is then used as the anomaly detection. The predicted faulty data, captured by autoencoder, are put into the LSTM network to identify the types of faults. It basically combines the strong low-dimensional nonlinear representations of the autoencoder for the rare event detection and the strong time series learning ability of LSTM for the fault diagnosis. The proposed approach is compared with a deep convolutional neural network approach for fault detection and identification on the Tennessee Eastman process. Experimental results show that the combined approach accurately detects deviations from normal behaviour and identifies the types of faults within the useful time.

## 1. Introduction

Modern industrial control systems deal with multivariate time series data of multiple correlated signals between sensors and actuators [1,2,3,4]. Fault detection and diagnosis (FDD) has tremendous potential to improve the operational reliability and stability of industrial processes since the objective of FDD is to minimize the production losses, while ensuring the safety of human and equipment [5]. FDD identifies anomalies of critical equipment by analyzing and mining recorded data to deliver notifications to operators [6,7]. Recent embedded systems, wireless networks and cloud computing provide a strong technology push for the development of next generation FDD systems [3,4,8,9].

Since industrial monitoring data have become much larger as both the number of samples and the dimensionality increased, traditional model- or knowledge-based approaches, involving extensive human intervention, are becoming too difficult to implement [6]. Finding abnormal measurements is extremely challenging due to the complex nonlinear relationships among the signals [1]. Furthermore, industrial faults rarely occur during the stable operation of the control processes such as machine failures in manufacturing [1,10]. While several feature extraction and classification methods have been proposed for fault detection and analysis problems [11,12], the fault classification accuracy is quiet poor for practical industrial processes [13]. Most deep learning techniques are not able to efficiently solve an overfitting problem due to the severely unbalanced samples of rare events [12]. Furthermore, FDD must provide timely detection and diagnosis of abnormal situations to avoid adverse consequences [6,10]. While the real-time FDD is one of the critical issues in practice, the time delay is not well investigated in previous research. In summary, the major technical challenges of the industrial FDD problem are the high complexity between signals, the unbalanced samples of faulty events and the time delay of fault detection and identification.

Prior knowledge can help avoid such problems by combining different machine learning techniques. In this paper, we propose an integrated learning approach for jointly achieving fault detection and fault diagnosis of rare events in multivariate time series data. The proposed approach combines an autoencoder to detect rare events and a long short-term memory (LSTM) network to identify the types of faults. The simple autoencoder trains the model with only normal data and evaluates input variables to detect rare faults for anomaly detection. The faulty data, detected by autoencoder, are put into LSTM network to identify their types. The proposed approach of FDD is evaluated on the Tennessee Eastman benchmark [13].

The rest of the paper is organized as follows. Section 2 discusses the related works of the FDD problem. Section 3 describes the architectures and algorithms of our integrated approach based on autoencoder and LSTM network. Section 4 illustrates the evaluation setup in detail. In Section 5, we present temporal fault detection and fault diagnosis results on the benchmark dataset of the proposed technique. Section 6 summarizes this paper.

## 2. Related Works

FDD is an active research field that has stimulated the development of a broad range of methods and heuristics for industrial processes [1,5,13]. Traditional approaches extract the low-dimensional feature from the high-dimensional raw data and then classify the faults for fault detection or fault diagnosis based on the feature space [12]. Most well-known feature extraction methods for the FDD problem are principal component analysis (PCA) [14,15], independent component analysis (ICA) [16] and partial least squares (PLS) [17]. PCA uses the orthogonal transformation to convert high-dimensional correlated variables into linearly uncorrelated variables in a new coordination such that the variance is maximized. PCA-based approaches have been used for the industrial fault detection problem [14,15]. ICA extracts independent non-Gaussian components from multivariate data by maximizing the statistical independence of the predicted components [18,19]. ICA generally performs better than PCA for non-Gaussian processes [20].

However, both PCA and ICA have the fundamental limitations to extract nonlinear features since these methods rely on linear technique. The kernel tricks are applied to convert the raw nonlinear data into a linear feature space such as kernel PCA (KPCA) [21] and kernel ICA (KICA) [22]. These methods generally have the high computational complexity due to its kernel operations if the training dataset is large. Furthermore, the performance of both KPCA and KICA is sensitive to the kernel hyperparameters. Hence, the kernel extension for both PCA and ICA is not a efficient solution for the real-time fault detection of industrial processes. A comparative research on these classical techniques for FDD is performed on the industrial benchmark of the Tennessee Eastman process (TEP) [13]. However, the traditional feature extraction methods only achieve the classification accuracy less than 50% on the TEP dataset.

Different fault diagnosis methods use the extracted features to find the root cause. The fault diagnosis task is a supervised learning problem where the objective is to correctly assign a sample to one of the fault classes. Different classification methods such as support vector machines [23,24], Bayesian discriminant functions [25] and neural networks [26] are used for the fault diagnosis. Numerous studies show that one of the promising options is the multilayer perceptron of neural networks to extract features and identify the types of faults [26,27,28]. However, the conventional classifiers based on the multilayer perceptron do not essentially capture the temporal information of the dynamical behavior of faults in industrial processes. In fact, these static networks discard the temporal correlation of multivariate variables since each data is independently used to train the network.

During last years, the deep learning technique achieves the outstanding performance in various application fields [29]. Some deep learning solutions are recently applied for the industrial fault diagnosis. In Reference [30], a hierarchical deep neural network is proposed to control multiple deep neural networks by a supervisory agent. After various types of faults is clustered into few groups, a specific deep neural network is trained for each group. The supervisory neural network triggers the specific deep neural network dependent on the input variables. The hierarchical network achieves the average classification accuracy around 80.5% on the TEP dataset.

Recurrent Neural Network (RNN) [31] and its variants are developed to deal with complex sequential problems [32,33]. In particular, the LSTM network effectively extracts the long-term temporal dependencies along with the short-term ones for time series data by using nonlinear gates. In Reference [34], LSTM is used to estimate the remaining useful lifetime along with the probability of fault occurrence under the complex operation mode and hybrid degradation for aircraft engine products. It is also used for timely detection and diagnosis of faults in railway track circuits [35]. The results show that the LSTM network learns the spatial and temporal dependencies to characterize the types of faults from the electrical current measurements.

## 3. Combined Autoencoder and LSTM Network

Our objective is to identify the types of faults of severely unbalanced data for practical industrial processes. The basic idea is to separate the detection problem to determine rare events and the diagnosis problem to identify the types of rare fault events. This section describes how to deal with multivariate time series data for FDD using autoencoder and LSTM network.

Consider a time series $x=\left\{x_{1},x_{2},\ldots ,x_{N}\right\}$, where each point $x_{t}\in R^{\mathrm{dx}}$ is a dx-dimensional input vector in the time series. Each sample is labeled as normal or fault type according to the records of multivariate processes. The measured variables typically have substantially different scales [1,10]. To recenter training data, each training sample is subtracted by the per-feature mean and divided by the standard deviation of the whole training samples. The normalization results in zero mean value and unit standard deviation data to efficiently train the network.

**Figure 1:**
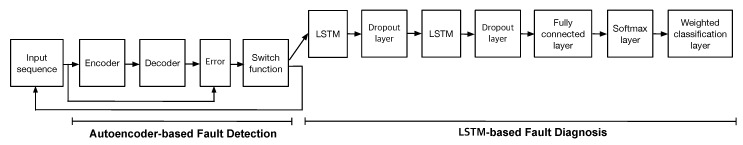
Structure of combined autoencoder and long short-term memory (LSTM) network for fault detection and fault diagnosis.

Figure 1 describes the core components of the proposed architecture that combines autoencoder and LSTM network for FDD. The normalized data are used to train the proposed model. The network starts with a sequence input layer of the multivariate time series samples. The autoencoder then analyzes the time series data to detect rare events using the concept of anomaly detection. Note that an autoencoder is particularly useful to extract nonlinear features for unsupervised learning from various types of data [14,36,37]. Once a fault is detected, the LSTM network learns the dependencies between various time steps of sequential data to identify the types of faults. LSTM has been applied for complex sequential problems since it is particularly efficient to learn the long-term dependencies of unknown lengths of time series data by using the nonlinear gating functions [38,39]. Instead of traditional parameter tuning using a backpropagation algorithm, we simply combine two trained models of both autoencoder and LSTM network where each model is separately trained. This approach has proven to be very efficient since it considerably improves the convergence rate of the overall structure with less numerical issues [29].

### 3.1. Autoencoder-Based Anomaly Detection

The practical industrial dataset is severely unbalanced where the positively labeled data are around 5–10% of the total [1,5,10]. In a rare event problem, most deep learning techniques are fundamentally limited by the amount of positively labeled data. This severely affects the precision and recall of a classification model [12]. Traditional dropout and batch normalization do not work well for such severely unbalanced dataset. Furthermore, the undersampling approach to balance the dataset has the fundamental limitation of a low accuracy [32].

We first learn a prediction model using the autoencoder [28,40] and then detect anomalies according to the reconstruction error. We divide the multivariate time-series data into two parts: positively labeled and negatively labeled. The negatively (resp. positively) labeled data is the normal (resp. faulty) state of the process. The autoencoder basically extracts the features of the normal state to perform rare event classification. It is trained only using the negatively labeled data as the anomaly detection since we consider the extremely rare events with less than 5–10% positively labeled data.

In Figure 1, the autoencoder is composed of two modules: encoder and decoder. The encoder converts high-dimensional multivariate input data into low-dimensional features, which is then converted back to the input data by the decoder. The autoencoder with three layers, namely, one input layer, one hidden layer and one output layer, is considered for simplicity in this paper.

If the input to the autoencoder is a vector $x_{t}\in R^{\mathrm{dx}}$, then the encoder maps the vector $x_{t}$ to another vector $m_{t}\in R^{\mathrm{da}}$ and the decoder maps the encoded representation $m_{t}$ back into the estimated vector ${\hat{x}}_{t}$ of the original input vector as follows:

(1) $$\begin{array}{cc} m_{t} & =\sigma_{1}\left(W_{1}x_{t}+b_{1}\right) \end{array}$$

(2) $$\begin{array}{cc} {\hat{x}}_{t} & =\sigma_{2}\left(W_{2}m_{t}+b_{2}\right) \end{array}$$

where $\sigma_{1}$ and $\sigma_{2}$ are the activation function and the subscript (1) and (2) indicate the hidden and output layer, respectively. The feature is typically in a reduced dimension da<dx [41]. The hyperbolic tangent function is used as the activation functions. The weight matrix $W_{1}\in R^{\mathrm{da}\times \mathrm{dx}}$ (resp. $W_{2}\in R^{\mathrm{dx}\times \mathrm{da}}$) and bias vector $b_{1}\in R^{\mathrm{da}}$ (resp. $b_{2}\in R^{\mathrm{dx}}$) are the learnable parameters for the encoder (resp. decoder).

The standard autoencoder learns only a model in which the output takes the same value as the input data. This process requires that the model learns the regularized features and that it can output data which efficiently ignore trivial noise or unknown faulty behavior. Our target is not only to minimize the training error between the input $x_{t}$ and the reconstructed output ${\hat{x}}_{t}$ but also to address the overfitting issue with additional $L_{2}$ regularization term of the weights. Hence, the regularized cost function is

(3) $$\begin{array}{cc} J(W_{1},W_{2},b_{1},b_{2}) & =\sum_{t=1}^{N}\frac{1}{2}∥x_{t}-{\hat{x}}_{t}{∥}_{2}^{2}+\frac{\lambda }{2}\left(∥W_{1}{∥}_{2}^{2}+{∥W_{1}∥}_{2}^{2}\right) \end{array}$$

where *t* indicates the *t*-th training sample, $\left\{W_{1},W_{2},b_{1},b_{2}\right\}$ are the decision parameters and λ is the coefficient of the regularization term. The value of the coefficient λ determines a trade-off between the training error and the generalization ability.

We learn the parameters $\left\{W_{1},W_{2},b_{1},b_{2}\right\}$ by using a backpropagation algorithm [40]. For fault detection and analysis, we compute ${\hat{x}}_{t}$ by Equations (1) and (2). Then the reconstruction error vector $e_{t}=x_{t}-{\hat{x}}_{t}$ is used to calculate the root-mean-square error (RMS) as an anomaly score. A well-trained autoencoder predicts any new data that is coming from the normal state of the process since it will have the same pattern or distribution. Therefore, the reconstruction error will be small. However, the autoencoder results the high reconstruction error if we try to reconstruct a data from a rare event. An observation $x_{t}$ is classified as “anomalous” if rms(e)>θ, else the observation is classified as “normal”. In Section 5, the decision threshold θ is optimized based on the number of true positives and false positives of the benchmark dataset.

### 3.2. LSTM-Based Fault Diagnosis

Figure 1 describes the flow of a time series data through the LSTM-based classifier. Once a fault is detected through the autoencoder, then time series data is put into the LSTM-based classifier for the fault diagnosis. Note that time series data is labeled with their corresponding fault classes, including normal data. The input data of LSTM includes the previous time series data before the first event of the fault detection to compensate the time delay and the false positives of the anomaly detection. The LSTM-based fault diagnosis consists of two phases: feature extraction and classification. We use two LSTM layers to transforms the multivariate data into a high level representation as the feature extraction. The dropout layer is used after each LSTM layer to prevent overfitting. Then, a fully connected layer, a softmax layer and a weighted classification layer are used to assign the transformed representation to one of the fault classes, as shown in Figure 1.

**Figure 2:**
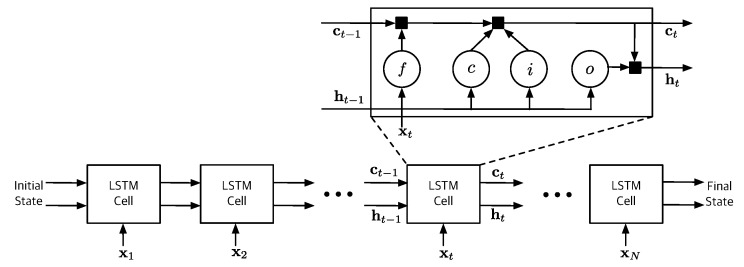
Structure of standard LSTM network.

Figure 2 illustrates the typical LSTM structure consisting with a number of cells [33]. The cell computes the hidden state $h_{t}\in R^{\mathrm{dh}}$ and the updated cell state $c_{t}\in R^{\mathrm{dh}}$ based on the previous state $\left(c_{t-1},h_{t-1}\right)$ and the sequential input $x_{t}$ at time step *t*. Note that the first cell uses the initial states $\left(c_{0},h_{0}\right)$. Each cell of the LSTM network is regulated by three nonlinear gate units, namely, input gate, forget gate, and output gate. These gate units are the essential components to learn the long-term patterns by preventing memory contents from irrelevant inputs and outputs.

The main recurrent transitions between LSTM cells are

(4) $$\begin{array}{cc} z & =Wx_{t}+Rh_{t-1}+b \end{array}$$

(5) $$\begin{array}{cc} c_{t} & =\sigma_{g}\left(z_{t}^{f}\right)⊙c_{t-1}+\sigma_{g}\left(z_{t}^{i}\right)⊙\sigma_{s}\left(z_{t}^{c}\right) \end{array}$$

(6) $$\begin{array}{cc} h_{t} & =\sigma_{g}\left(z_{t}^{o}\right)⊙\sigma_{s}\left(c_{t}\right) \end{array}$$

where

(7) $$\begin{array}{c} z={\left[\begin{array}{cccc} z_{t}^{i} & z_{t}^{f} & z_{t}^{c} & z_{t}^{o} \end{array}\right]}^{⊺}\in R^{4\mathrm{dh}}\;, \end{array}$$

⊙ denotes the Hadamard product, i,f,c and *o* indicate the input gate, forget gate, cell candidate and output gate, respectively. The hyperbolic tangent function and sigmoid function are used as the state activation function $\sigma_{s}$ and gate activation function $\sigma_{g}$, respectively. The learnable parameters of the input weights W, the recurrent weights R and the bias vector b are concatenated as follows:

(8) $$\begin{array}{cc} W & ={\left[\begin{array}{cccc} W_{t}^{i} & W_{t}^{f} & W_{t}^{c} & W_{t}^{o} \end{array}\right]}^{⊺}\in R^{4\mathrm{dh}\times \mathrm{dx}}\;, \end{array}$$

(9) $$\begin{array}{cc} R & ={\left[\begin{array}{cccc} R_{t}^{i} & R_{t}^{f} & R_{t}^{c} & R_{t}^{o} \end{array}\right]}^{⊺}\in R^{4\mathrm{dh}\times \mathrm{dx}}\;, \end{array}$$

(10) $$\begin{array}{cc} b & ={\left[\begin{array}{cccc} b_{t}^{i} & b_{t}^{f} & b_{t}^{c} & b_{t}^{o} \end{array}\right]}^{⊺}\in R^{4\mathrm{dh}}\;. \end{array}$$

To solve the overfitting problem of fault diagnosis, the neurons stop working with probability p=0.2 as the basic dropout method in each dropout layer of Figure 1 [42].

A fully connected layer multiplies the output of the LSTM network with the weight matrix $W_{c}\in R^{\mathrm{dc}\times \mathrm{dh}}$ and adds the bias vector $b_{c}\in R^{\mathrm{dc}}$. Hence, it returns

(11) $$\begin{array}{c} p_{t}=W_{c}h_{t}+b_{c} \end{array}$$

where $p_{t}\in R^{\mathrm{dc}}$ and dc is the number of fault classes including the normal state. The output of the fully connected layer is then put into a softmax regression layer for the multi-class classification. It gives a output vector with a total number of fault classes where each element predicts the probability of one of the fault classes [43].

Finally, the classification layer assigns each input data to the one of fault classes based on the output of the softmax function. Since each fault class may occur with different frequencies, a weighted classification layer is used to compute the weighted cross entropy loss for classification problems. For prediction scores and training targets, the weighted cross entropy loss function is

(12) $$\begin{array}{c} J\left(W,R,b,W_{c},b_{c}\right)=-\frac{1}{N}\sum_{t=1}^{N}\sum_{j=1}^{\mathrm{dc}}\omega_{i}I_{tj}\log\left(y_{tj}\right) \end{array}$$

where $\omega_{i}$ is a weight of class *i*, $I_{tj}$ is the indicator function and $y_{tj}$ is the value from the softmax function for *t*-th sample to class *j*. The weights of different classes ω are estimated using the whole training dataset.

The dataset is labeled with different fault classes to support a supervised learning task. We use the mini-batch stochastic-gradient algorithm based on the estimated lower-order moments to solve the optimization problem [32,44]. Training data samples are divided into mini-batches. Note that all sequences of each mini-batch must have the same length. Since the sequence length may depend on the fault type in practice, we first sort the training data by its length. We then pad the extra data to have the same sequence length in each mini-batch.

## 4. Evaluation Setup

In this section, we describe the benchmark dataset of the practical industrial process and the existing deep neural network approach that we used to compare our proposed method.

### 4.1. Tennessee Eastman Challenge Problem

We evaluate the performance of the proposed method for FDD on Tennessee Eastman Process (TEP) [45]. TEP is a widely-used benchmark testbed to investigate the large-scale control and FDD schemes of realistic chemical processes [13]. The simulation data of TEP are highly nonlinear with strong coupling and dynamical behavior. The simulation code and data are available for download in References [46,47], respectively.

**Figure 3:**
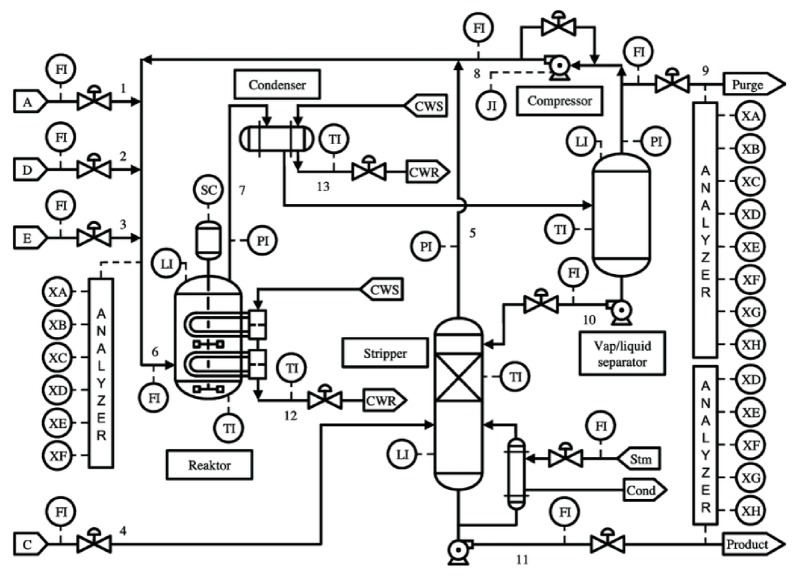
Diagram of the Tennessee Eastman Process (TEP) simulation [45]. Reproduced with permission from J.J. Downs, Computers & Chemical Engineering; published by Elsevier, 1993.

The main structure of the TEP simulator is described in Figure 3. The TEP produces two products *G* and *H* from four reactants A,C,D,E with additional byproduct *F*. The reactions are

$$\begin{array}{cc} A_{\left(g\right)}+C_{\left(g\right)}+D_{\left(g\right)} & \to G_{\left(\mathrm{liq}\right)}\;,\;\;\mathrm{Product}\;1\\ A_{\left(g\right)}+C_{\left(g\right)}+E_{\left(g\right)} & \to H_{\left(\mathrm{liq}\right)}\;,\;\;\mathrm{Product}\;2\\ A_{\left(g\right)}+E_{\left(g\right)} & \to F_{\left(\mathrm{liq}\right)}\;,\;\;\;\;\mathrm{Byproduct}\\ 3D_{\left(g\right)} & \to 2F_{\left(\mathrm{liq}\right)}\;,\;\mathrm{Byproduct} \end{array}$$

All chemical reactions are irreversible, exothermic and approximately first-order with respect to the reactant concentrations. The reaction rates are a function of temperature through an Arrhenius expression. The reaction to produce *G* has a higher activation energy than the one producing *H*, thus resulting in more sensitivity to temperature.

To model a practical industrial process, the TEP simulator consists of five major units: reactor, condenser, compressor, separator and stripper. The gaseous reactants are fed into the reactor where liquid products are formed. The product stream of the reactor is cooled through a condenser and fed to a vapor-liquid separator. Non-condensed components are recycled back to the reactor via a compressor. Condensed components are moved to a product stripping column by stripping with feed stream number 4 to eliminate remaining reactants. Products *G* and *H* are separated in a downstream refining section from the stripper base. The inert and byproducts are purged as vapor from the vapor-liquid separator. In TEP, we monitor a total of 52 variables including 41 measured variables and 11 manipulated variables [45].

The modern industrial systems interact with multiple subcomponents where each component has several different failure modes [48,49]. Furthermore, each failure mode typically has long-term dependencies along with short-term ones of time series data. Besides normal data, Table 1 describes 20 different types of faults to evaluate various FDD methods.

**Figure 4:**
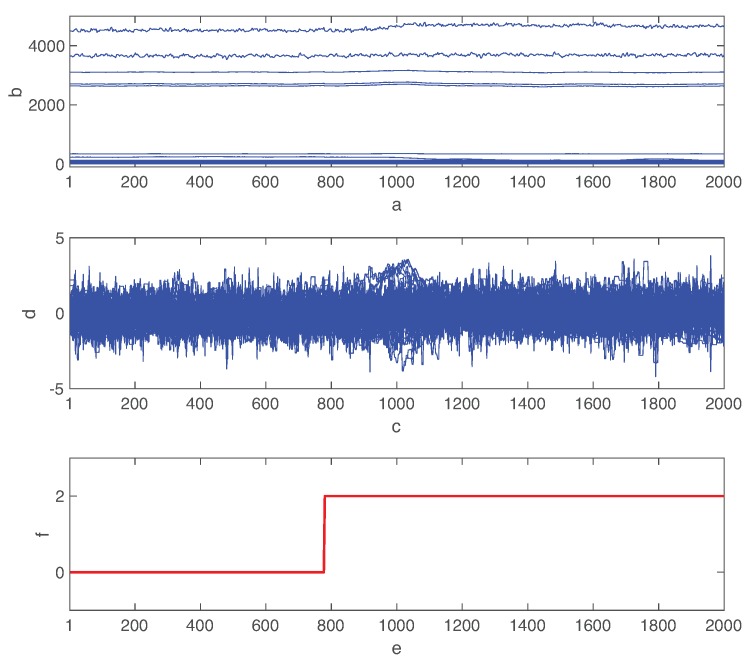
Process state measurements when fault 02 occurs at 780min.

Figure 4 shows the raw process variable deviations from their normal states when fault 02 is introduced at 790min after the simulation started. We also show the normalized variables by the mean and standard deviation of each feature. Although the time-varying features of multivariate data are critical to identify the types of faults, the distinction between various types of faults is a challenging task due to the complex interaction among control processes [50]. In fact, the fault effect is considerably different for various process variables. Furthermore, even if some variables are oscillating due to faults, there is a significant delay to recognize it as shown in Figure 4. The combined autoencoder and LSTM network must mine the hidden features of time series data. More detailed information of TEP is described in Reference [45].

The simulation starts to run in the normal state for 110h. The specific fault out of 20 different types is then injected and it continues to run for 10h. Hence, the total simulation time is 120h based on the recommendation of the TEP model [45]. Each simulation of the fault repeats 500 times with various initial states and random noise. We set the sampling time as 3min (20 samples/h) to support the fast fault detection and fault diagnosis [51]. We randomly select 80% time series sample as training sets and the remaining sample as testing sets. We only use the normal state samples without any faults to train the autoencoder for fault detection where anomalous and normal states correspond to positive and negative class, respectively. In addition, 20% of the whole training set is used as the validation set to optimize the decision threshold θ for the autoencoder. On the other hand, the time series samples with temporal features are used to train the LSTM-based classifier. In this training set, we remove the first 109h of the normal state samples in each simulation. Hence, each training set of the LSTM network consists with 1h of normal data and 10h of faulty state data. The fraction of the normal state samples prior to faults is used to compensate the detection delay and the possible false positives of the autoencoder. The input sequence of the testing set for LSTM includes 2h time series data before the first event of the fault detection, captured by the autoencoder.

### 4.2. DCNN

In previous research, a deep convolutional neural network (DCNN)-based approach achieves the best reported results in multivariate time series data from the simulated TEP dataset [52]. We compare the fault diagnosis performance of LSTM and DCNN-based approaches on the TEP dataset.

Based on the recommendation of Reference [52], we build the DCNN-based approach with three convolutional layers, two max pooling layers and two fully connected layers. The multiple input samples with time steps are used to train the network. It basically concatenates *m* input vectors to m×n matrix where *m* is the number of samples and *n* is the number of variables per sample. Since the default length of the period is 1 h, the size of the input matrix is set to 20×52 [52]. The detailed architectures and parameters of the DCNN-based approach can be found in Reference [52].

## 5. Performance Evaluation

This section firstly evaluates the fault detection performance of the autoencoder on the TEP benchmark. We then compare the fault diagnosis performance of LSTM against the DCNN-based approach. We implement the proposed scheme and DCNN using the deep learning package of TensorFlow. Experiments are conducted on a personal computer with Inter Core i9-7920X CPU, 128 GB memory, and NVIDIA TITAN RTX GPU. We use not only the classical metrics such as accuracy, precision, recall, F-score but also time delay to evaluate the fault detection and fault diagnosis performance.

### 5.1. Fault Detection

**Figure 5:**
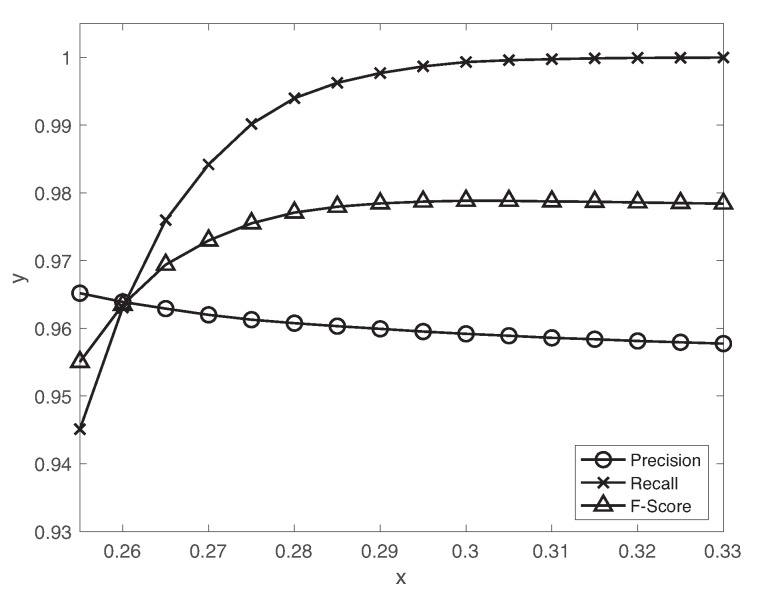
Precision, recall, F-score as a function of different decision thresholds θ=0.25,…,0.33 of the autoencoder.

Figure 5 presents precision, recall, F-score metrics as a function of different decision thresholds θ=0.25,…,0.33 of the autoencoder. Remind that we label the high reconstruction errors as an anomalous data dependent on the decision threshold. With the increase of the threshold value, the precision slightly decreases but the recall drastically increases. We set the decision threshold θ=0.3 to achieve a reasonable tradeoff between precision and recall using the validation set. The accuracy, precision, recall, F-score and false positive rate with θ=0.3 are 0.96,0.96,0.99,0.98,0.39, respectively. Since the fault detection is a rare event detection problem, F-score is mainly used as the suitable metric for evaluation. Hence, we achieve reasonably high performance using the autoencoder. Although the false positive rate is relatively high, the LSTM-based classifier effectively reduces the normal data miss-classified as fault data during the fault diagnosis as we will see in Section 5.2. We also evaluate the proposed scheme for the rare event detection of the realistic industrial data [10]. The autoencoder achieves the good performance in terms of accuracy 0.88, precision 0.88, recall 0.99, F-score 0.94 and false positive rate 0.99 with θ=0.1.

**Figure 6:**
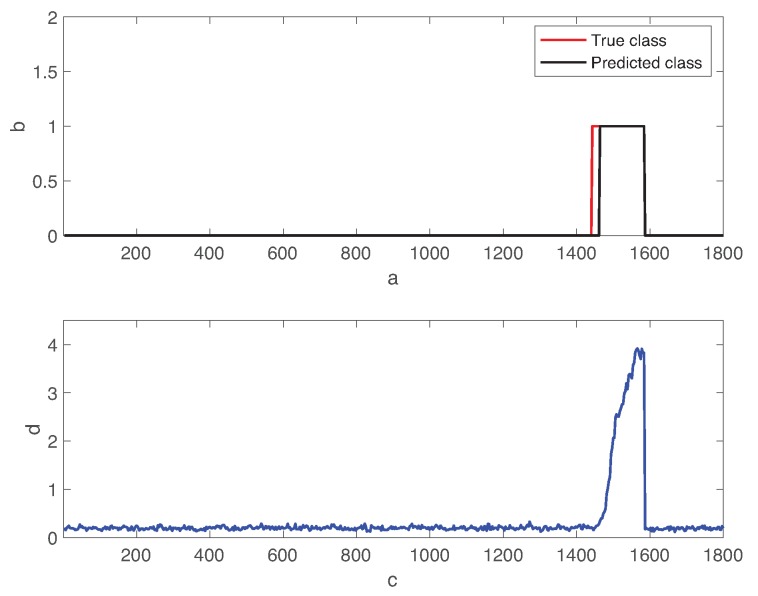
Transient behavior of the fault state and the residual error of the autoencoder. Fault 01 is introduced to the normal state at 1440min.

Figure 6 shows the transient behavior of the process state and the residual error of the autoencoder over time. In the figure, the red and black lines represent the true fault class and the predicted one, respectively. Fault 01 is introduced to the normal state at 1440min. The residual error is significantly increased in the faulty state than the one in the normal operation mode. The autoencoder predicts the fault state when the residual error is over the decision threshold θ=0.3.

In Figure 6, we observe the time delay of the fault detection. Detection delay is the time elapsed from the fault injection instance to the time at which the autoencoder provides the correct prediction and remains. Although the lower threshold value reduces the detection delay, it increases the risk of the false alarms. In fact, since the residual errors do not remain low during the normal operation mode, this increases the probability of normal condition data to be miss-classified as the fault positives.

**Figure 7:**
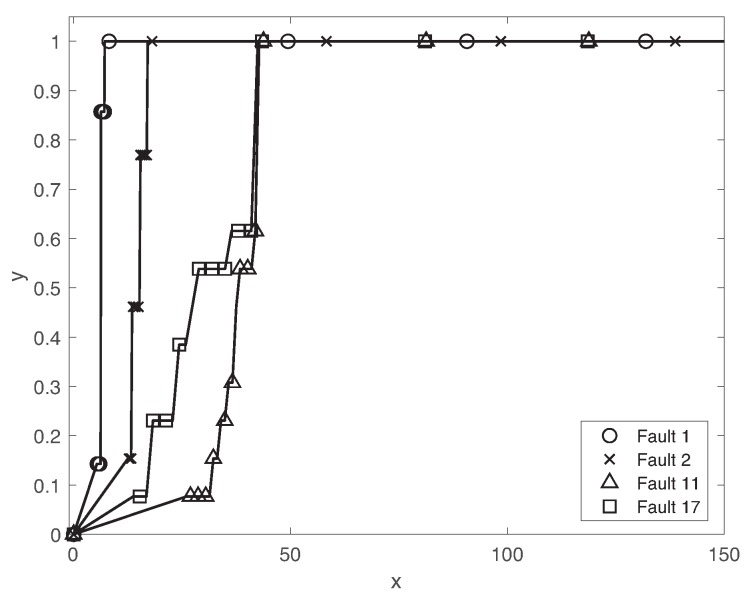
Cumulative density functions (CDFs) of fault detection delay of faults 01,02,11,17.

Since the fault propagation effect is a dynamical process, we investigate the transient behavior of the fault detection performance of the proposed method. Figure 7 illustrates the cumulative density function (CDF) of the fault detection delay of different faults 01,02,11,17. The fault detection delay clearly depends on the fault classes. While more than 90% of faults 01,02 is detected within 20min, the detection delays of other faults 11 and 17 are highly varying. One of the main reasons is the different dynamical effects of faults on the chemical processes. The effects of faults 11 and 17 are slowly propagated over the control systems. Furthermore, it is not possible to analyze all types of faults that can potentially happen in a system operated under different conditions and degradation levels due to complex interactions between the subsystems and the environment. The fault detection delay of faults 01,02,11,17 is comparable with the best results obtained by various algorithms in Reference [53].

**Figure 8:**
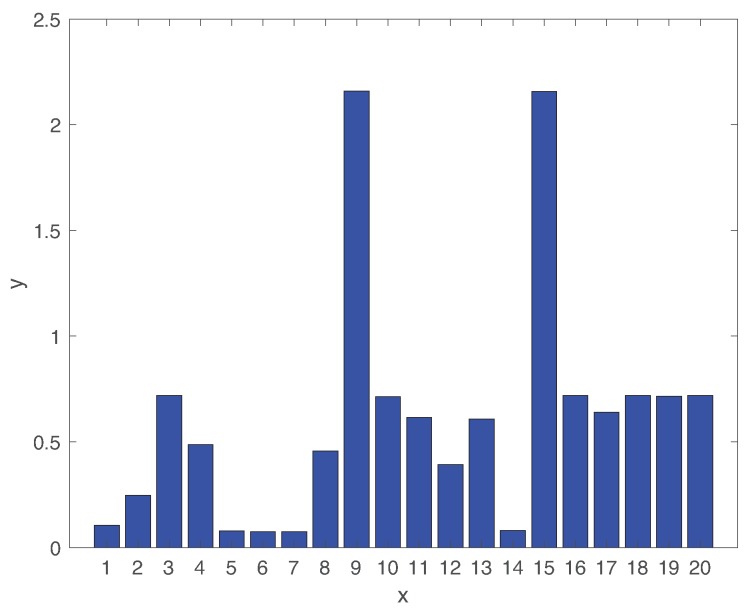
Expected fault detection delay of all types of faults.

Figure 8 shows the average detection delay of all types of faults. The detection delays are largely dependent on the types of faults. While the average detection delay of faults 01,02,05,06,07,12,14 is less than 30min, rest faults have relatively high delays. The detection delays of faults 09 and 15 are particularly high larger than 2h. In fact, these two fault classes are hard to identify in the TEP testbed [30,52], as we will discuss more details in Section 5.2.

### 5.2. Fault Diagnosis

The fault diagnosis performance of the LSTM-based classifier is compared to that of the DCNN-based one, once a fault is detected. We first use the confusion metrics, receiver operating characteristic (ROC) curves and area under the curve (AUC) to compare the overall performance between LSTM and DCNN. The diagnosis time is then explored to understand the transient performance.

For this fault diagnosis step, we first evaluate the confusion matrices of LSTM and DCNN approaches for all types of faults. A confusion matrix is a specific table to describe the efficiency of a classification model using a testing dataset. The actual confusion matrices for fault diagnosis are not included due to the space limit in the paper. The classification accuracies of LSTM and DCNN are 91.9% and 76.4%, respectively. LSTM gives significant improvement of 16.9% over DCNN in terms of average accuracy. However, some faults 03,09,15 are hard to diagnosis for both LSTM and DCNN approaches. In both approaches, the classification accuracies of these three faults are below 75%. The LSTM model shows that except faults 03,09,15, the other fault types are identified with more than 91% accuracy. In fact, our proposed combined autoencoder and LSTM outperforms the classical feature extraction methods and the hierarchical deep neural network [30]. Note that the classical dynamic feature extraction method and the hierarchical deep neural network achieve the classification accuracy less than 50% and 80.5% (except faults 03,09,15), respectively. On the other hand, the DCNN model gives poor classification accuracies for faults 03,04,05,09,15 less than 65%.

Let us discuss more details of specific faults 03 and 09 since both faults are hard to distinguish in the experimental results [30,45]. The main reason is that fault 03 produces a step change while fault 09 is random variation to the same D feed temperature at the top left corner of Figure 3. Hence, the time series samples of both faults are easily confused for the fault diagnosis. Furthermore, previous research shows that two faults 09 and 15 are also hard to classify in References [30,50,52]. Therefore, for convenience, we only consider all types of faults excluding two faults 09 and 15 in the rest of the paper.

By excluding these two faults, we have also analyzed the confusion matrices of LSTM and DCNN approaches for other 18 different types of faults. Both LSTM and DCNN approaches are well performing on identifying most fault classes since they get the high classification accuracies of 96.8% and 88.2%, respectively. Hence, the temporal patterns of various faults are mined through both LSTM and DCNN for the fault diagnosis. The classification accuracy of DCNN is less than 75% for faults 03,04,05. By contrast, LSTM identifies these faults with much higher accuracy of 91.7%, 98.9%, and 98.9%, respectively. The main reason is that these fault cases have long-term temporal dependence longer than 1 h. The worst classification accuracies using LSTM and DCNN are 91% and 65.5% for faults 18 and 03, respectively. These results prove that the LSTM-based classifier is an effective technique for the fault diagnosis since it adaptively learns the dynamical temporal information of various fault types using the nonlinear gates of each cell.

The classification accuracy using DCNN is lower than the one of the existing results [52]. Three variables out of 52 variables are excluded since they are constant in the simulation setup of Reference [52]. However, our classical simulation results show time varying measurements of all 52 variables. Hence, the dataset of the TEP simulation affects the accuracy of overall results.

**Figure 9:**
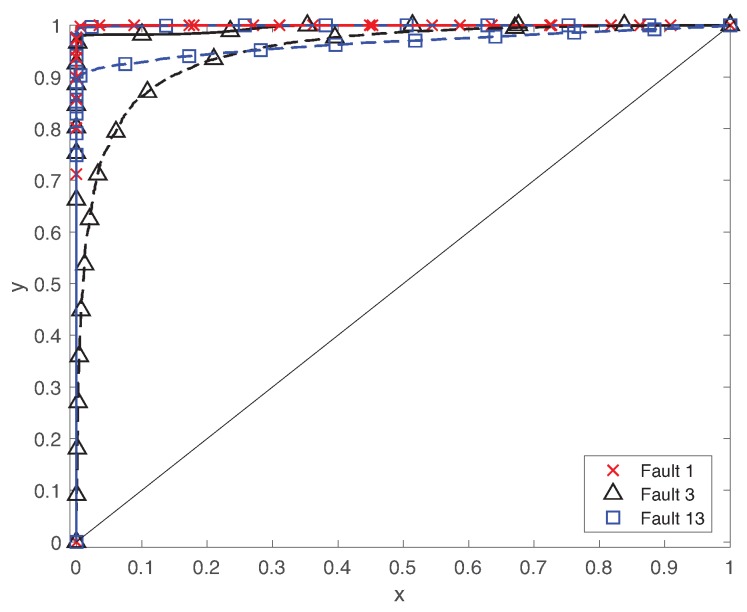
Receiver operating characteristic (ROC) curves using both LSTM and deep convolutional neural network (DCNN) for different faults 01,03,13.

Figure 9 shows the ROC curves using both LSTM and DCNN for different faults 01,03,13. The solid and dotted lines present the ROC curves using LSTM and DCNN, respectively. For each fault type, we plot the True Positive Ratio (TPR) against the False Positive Ratio (FPR) at different threshold values across the interval [0,1]. ROC curves move to the top right corner from the bottom left corner of the figure. The ideal operation point of the fault diagnosis is the top left corner where TPR = 100% and FPR = 0%. Hence, the diagnosis performance is better if the ROC curve gets to the top left corner. On the other hand, the overall performance is worse as the ROC curve is closer to the diagonal line of the figure. Since the LSTM-based classifier provides extremely low false positive rate, the ROC curve using LSTM is tightly closed to the top left corner. Hence, the proposed scheme considerably outperforms the ones of DCNN for faults 01,03,13. Furthermore, it effectively reduces the false positives of the autoencoder.

**Figure 10:**
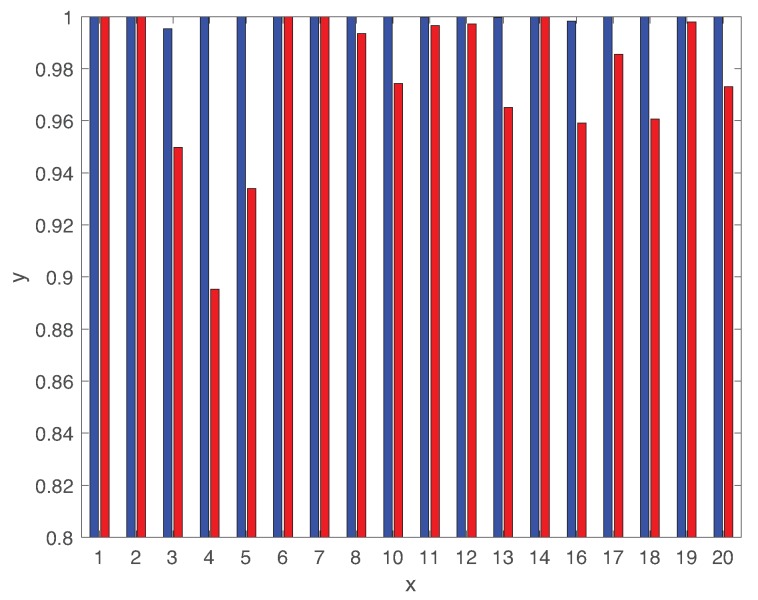
Area under the curve (AUC) using both LSTM and DCNN for 18 different types of faults.

To provide more detailed analysis, Figure 10 illustrates AUC of using both LSTM and DCNN for 18 different types of faults. We clearly observe that the LSTM-based classifier provides very high AUC closer to 1 with respect the ones using DCNN. The AUC value of DCNN is lower than 0.95 for faults 03,04,05. Hence, the comparison between LSTM and DCNN proves the benefits of the deeper structure of LSTM to capture the long-term temporal patterns along with short-term ones.

**Figure 11:**
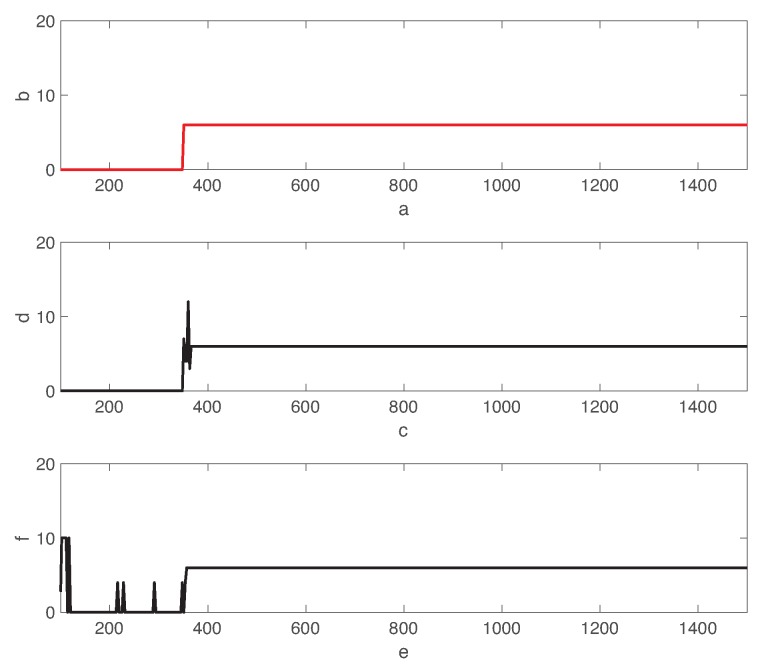
Transient behavior of the fault state and the prediction using LSTM and DCNN. Fault 06 is introduced to the normal state at 380min.

Now, we discuss the fault diagnosis delay of both LSTM and DCNN approaches. Figure 11 shows the time transient behavior of the actual fault state and predicted fault classes using LSTM and DCNN approaches. Both LSTM and DCNN approaches perform equally well to identify the fault type when fault 06 is injected at 380min. However, the transient behaviors between LSTM and DCNN are considerably different. While the LSTM-based classifier does not give any false positives during the normal state, the fault prediction is slightly oscillated to correctly estimate fault class 06. On the other hand, the DCNN-based approach gives some fault positives during the normal operation mode. Hence, we clearly observe the benefit of the LSTM network to reduce the false positives of the autoencoder. Remind that the DCNN-based approach gives the higher false positive rate as shown in Figure 9 and Figure 10.

In Figure 11, fault 06 using LSTM is miss-classified as faults 03,07,12 for a short period. Hence, the LSTM-based classifier has the time delay to stabilize the fault diagnosis. In fact, the DCNN-based approach provides better fault diagnosis delay than the one using the LSTM network for fault case 06.

**Figure 12:**
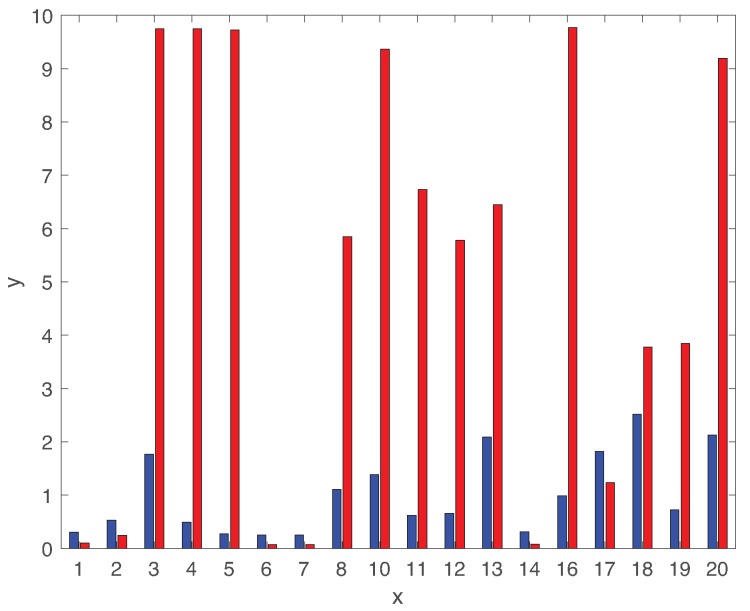
Expected fault diagnosis delay using LSTM and DCNN of 18 different types of faults.

Figure 12 compares the average fault diagnosis delay of both LSTM and DCNN for 18 different types of faults (except faults 09,15). It is interesting to observe that DCNN provides much lower diagnosis delay than the ones using the LSTM-based classifier for faults 01,02,06,07,14. However, the diagnosis delay using DCNN is significantly high for rest of fault classes. Hence, the diagnosis delays of DCNN are largely varying dependent on the fault class. By contrast, the diagnosis delay using LSTM is less sensitive to the fault class. Furthermore, the fault diagnosis delay using LSTM is comparable with the ones using various algorithms in Reference [53]. Figure 10 and Figure 12 show that the correlations between AUC and diagnosis delay of most types of faults are not clear for both LSTM and DCNN. Indeed, the AUC metric is not sufficient to characterize the diagnosis delay performance of various types of faults. Although none of these techniques give the optimal delay performance for all types of faults, the LSTM-based classifier provides better robustness to the various fault classes than the state of the art DCNN-based classifier.

## 6. Conclusions

This paper proposes a learning approach consisting of autoencoder and long short-term memory (LSTM) network for fault detection and diagnosis of rare events in a multivariate industrial process. A simple autoencoder trains the model with only normal data and evaluates multivariate time series data to detect rare faults for anomaly detection. The predicted faulty raw data are then put into the LSTM network to identify the types of faults. Two trained models of autoencoder and LSTM are combined to improve the convergence rate of the overall structure with less numerical issues. The Tennessee Eastman benchmark was then used to evaluate the fault detection and diagnosis performance. We analyzed its performances, not only the classical classification metrics but also the time delay for the fault detection and diagnosis. We demonstrated that the combined autoencoder and LSTM network accurately detects deviations from normal behaviour and identifies the fault type within useful time. Furthermore, the LSTM-based classifier provides the robust fault diagnosis with respect to the one using the existing deep convolutional neural network approach in various types of faults. The proposed approach achieves significant accuracy improvement of 16.9% over the deep convolutional neural network.

In practical industrial systems, a full coverage of representative data of all possible failures and their combinations is typically not available. For instance, existing fault detection and diagnosis approaches show that some fault classes of the Tennessee Eastman process are not possible to identify. Although some new sensors are possibly required to detect these faults for the predictive maintenance, the selection of the appropriate sensors is a challenging task. The future research work will be focused on bridging the gap between the physical control systems and the deep learning techniques to achieve predictive maintenance.

## Figures and Tables

**Table 1:** Fault description of the TEP simulation [45].

Fault Number	Description	Type
1	A/C feed ratio, *B* composition constant (Stream 4)	Step
2	*B* composition, A/C ratio constant (Stream 4)	Step
3	*D* feed temperature (Stream 2)	Step
4	Reactor cooling water inlet temperature	Step
5	Condenser cooling water inlet temperature	Step
6	*A* feed loss (Stream 1)	Step
7	*C* header pressure loss-reduced availablity (Stream 4)	Step
8	*A*, *B*, *C* feed composition (Stream 4)	Random variation
9	*D* feed temperature (Stream 2)	Random variation
10	*C* feed temperature (Stream 4)	Random variation
11	Reactor cooling water inlet temperature	Random variation
12	Condenser cooling water inlet temperature	Random variation
13	Reaction kinetics	Slow drift
14	Reactor cooling water valve	Sticking
15	Condenser cooling water valve	Sticking
16	Unknown	Unknown
17	Unknown	Unknown
18	Unknown	Unknown
19	Unknown	Unknown
20	Unknown	Unknown
